# Evaluation of a New Digital Automated Glycemic Pattern Detection Tool

**DOI:** 10.1089/dia.2017.0180

**Published:** 2017-11-01

**Authors:** María José Comellas, Emma Albiñana, Maite Artes, Rosa Corcoy, Diego Fernández-García, Jorge García-Alemán, Beatriz García-Cuartero, Cintia González, María Teresa Rivero, Núria Casamira, Jörg Weissmann

**Affiliations:** ^1^ Roche Diabetes Care Spain SL, Barcelona, Spain.; ^2^ Vithas Hospital Internacional Medimar, Paediatrics Unit, Alicante, Spain.; ^3^ Adelphi Spain, Barcelona, Spain.; ^4^ Hospital de la Santa Creu i Sant Pau, Endocrinology and Nutrition Department, Medicine Department, Universitat Autònoma de Barcelona, Barcelona, Spain.; ^5^ CIBER-BBN, Zaragoza, Spain.; ^6^ Hospital Universitario Virgen de la Victoria, Endocrinology and Nutrition Unit, Málaga, Spain.; ^7^ Hospital Universitario Ramón y Cajal, Pediatric Endocrinology and Diabetes Unit, Madrid, Spain.; ^8^ Complejo Hospitalario Universitario de Ourense, Endocrinology and Nutrition Unit, Ourense, Spain.; ^9^ Roche Diabetes Care Deutschland GmbH, Mannheim, Germany.

**Keywords:** Blood glucose, Pattern analysis, Diabetes, Pattern management, Automated tool

## Abstract

***Background:*** Blood glucose meters are reliable devices for data collection, providing electronic logs of historical data easier to interpret than handwritten logbooks. Automated tools to analyze these data are necessary to facilitate glucose pattern detection and support treatment adjustment. These tools emerge in a broad variety in a more or less nonevaluated manner. The aim of this study was to compare eDetecta, a new automated pattern detection tool, to nonautomated pattern analysis in terms of time investment, data interpretation, and clinical utility, with the overarching goal to identify early in development and implementation of tool areas of improvement and potential safety risks.

***Methods:*** Multicenter web-based evaluation in which 37 endocrinologists were asked to assess glycemic patterns of 4 real reports (2 continuous subcutaneous insulin infusion [CSII] and 2 multiple daily injection [MDI]). Endocrinologist and eDetecta analyses were compared on time spent to analyze each report and agreement on the presence or absence of defined patterns.

***Results:*** eDetecta module markedly reduced the time taken to analyze each case on the basis of the emminens eConecta reports (CSII: 18 min; MDI: 12.5), compared to the automatic eDetecta analysis. Agreement between endocrinologists and eDetecta varied depending on the patterns, with high level of agreement in patterns of glycemic variability. Further analysis of low level of agreement led to identifying areas where algorithms used could be improved to optimize trend pattern identification.

***Conclusion:*** eDetecta was a useful tool for glycemic pattern detection, helping clinicians to reduce time required to review emminens eConecta glycemic reports. No safety risks were identified during the study.

## Introduction

HbA1c is the gold standard as a summary of glycemic control, but regular self-monitoring of blood glucose (SMBG) is the most accessible way to monitor glycemic excursions. However, to adjust the treatment in an appropriate way, an accurate interpretation of the patients' reports is needed, but it is hindered by the large amount of data generated.

**FIG. 1.**
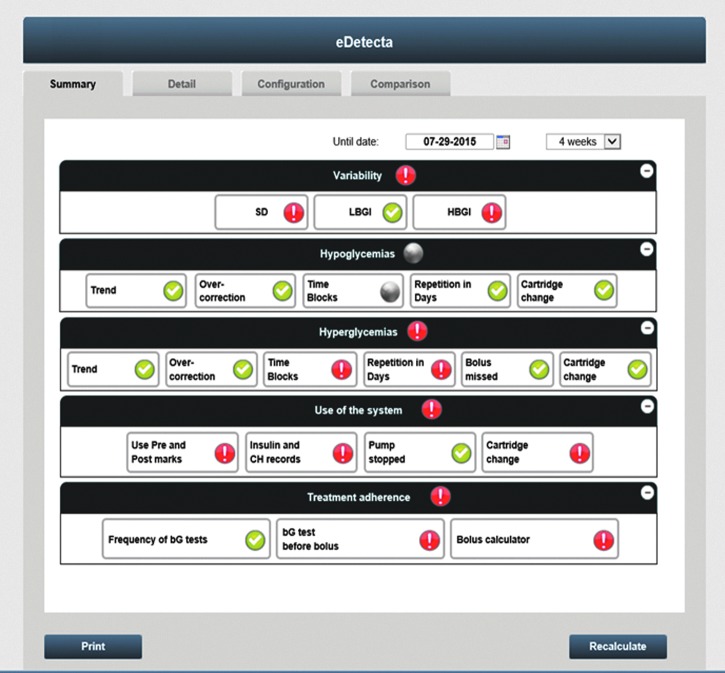
eDetecta dashboard.

Decision-making based on glycemic data is time-consuming and many parameters need to be considered. Pattern management has been defined as the systematic interpretation of SMBG data over time to determine whether changes are required to optimize blood glucose control.^1^ However, patterns are not always easy to detect or interpret and on-meter and web-based tools have been developed to support both patients and clinicians to better interpret the data collected.^2^ Emminens eConecta^®^ is a web-based tool that aims at personalized management of diabetes, where data collected in the devices can be downloaded and reviewed by healthcare professionals (HCPs). Clinicians typically review daily data and trend graphs from the last 4 registered weeks in combination with the electronic logbook and lists to fully understand and match glycemic, carbohydrate, and insulin data coming from pumps and bolus calculators. eDetecta is an emminens eConecta module developed to perform an automatic detection of glycemic patterns based on glycemic, insulin, and carbohydrate data; Figure 1 shows the eDetecta dashboard. The eDetecta module can analyze up to 22 patterns, grouped into 5 blocks: glycemic variability, hypoglycemia, hyperglycemia, use of the system, and adherence to treatment.

Definitions of every pattern are clearly specified in the platform and default values can be easily customized by HCP to allow a personalized pattern analysis. Default configuration was agreed by consensus among a group of endocrinologists and pediatric experts in diabetes.^3^

eDetecta can analyze data provided by glucose meters, bolus calculators, and insulin pumps. An easy-to-interpret dashboard shows if patterns are present (red) or absent (green).

The aim of this study was to compare time requirements and agreement in pattern detection between the automated eDetecta module and nonautomated analysis of the data reports by HCP.^4^

## Methods

### Study design

This multicenter, cross-sectional web-based evaluation included 37 endocrinologists from Spain and Portugal, with an average of 17 years of experience in diabetes. Participants were asked to analyze 4 cases, obtaining a maximum of 145 evaluations; we esteemed it would provide a reasonable statistical power.

The evaluation was organized in three parts: a preliminary questionnaire on eDetecta initial experiences (having used eDetecta in usual clinical practice with at least four patients), an evaluation of reports of real-life data downloads, and a final questionnaire on current barriers related to data analysis in clinical practice.

In the second part of the evaluation, participants were asked to analyze the glycemic patterns of four anonymized reports of real-life patients' data and complete a questionnaire indicating if the patterns available in eDetecta were present or not in the cases. The definition of the eDetecta and all the relevant information needed to perform manual pattern detection were available. The reports had been previously obtained from emminens eConecta platform and included data from two patients using insulin pumps (continuous subcutaneous insulin infusion [CSII] therapy) and two patients using a bolus calculator and multiple daily injections (MDI) to detect different patterns. These reports consisted of tendency graphs, data lists, as well as digital patient logbooks from a period of 4 weeks, representing the reports commonly obtained from the emminens eConecta. Table 1 summarizes the report content for each selected case and an example of the reports obtained, corresponding to the CSII case 2 (Supplementary Data; Supplementary Data are available online at www.liebertpub.com/dia). To minimize any potential bias due to the order in which cases were reviewed, six sequences of the four reports were generated and every participant was randomly assigned to one of them. The access period to the web-based evaluation lasted 4 weeks.

**Table 1.** Emminens eConecta Reports Content and Time Recovered for Each of the Four Cases Used to Evaluate the eDetecta Module

	*Graphs*				*Lists*			
	*Days*	*Weeks*	*Daily trend*	*Trend*	*Statistics*	*Combined*^a^*data (pages)*	*Combined*^a^*logbook (pages)*	*Time (weeks)*
Case 1 CSII	Half page	Half page	One page	Half page	One page	42	7	4
Case 2 CSII	Half page	Half page	One page	Half page	One page	27	4	2
Case 1 MDI	Half page	Half page		Half page	One page	10	6	4
Case 2 MDI	Half page	Half page		Half page	One page	8	5	4

^a^ Combined: glycemic, CH, insulin and device data at the same list.

CH, carbohydrate; CSII, continuous subcutaneous insulin infusion; MDI, multiple daily injections.

Time spent by participants to analyze each case and answers on presence or absence of predefined patterns were recorded. The clear definition of every pattern was available in the questionnaire.

### Data collection

Total time spent per participant to analyze every report consisted of two separate times: (1) time needed to read the report and (2) time needed to evaluate the presence or absence of patterns and answer the corresponding questions. Time spent by eDetecta on the automated analysis for each one of the four reports was also registered. All times were measured in minutes. Participant responses on the presence of patterns were compared with automated detected patterns by eDetecta. The questionnaire on current barriers related to data analysis in clinical practice included a total number of 30 questions, most of which were based on a 5-point score Likert-type scale (i.e., 1 is “not reliable at all” and 5 is “very reliable”).

### Statistical analysis

The level of agreement in pattern detection between HCPs and eDetecta was evaluated using Cohen's kappa coefficient (κ) and associated 95% confidence intervals (CIs) that were calculated both overall and for each of the four cases. A κ value of 0.81–1.00 indicates almost perfect agreement; a kappa coefficient of ≤0.2 indicates slight or no agreement. The analysis was adjusted by sequence case and country.^5^

The report reading time and patterns evaluation time of the four emminens eConecta reports by HCP and eDetecta module were evaluated. The total time spent to analyze each case (report reading time+pattern evaluation time) was calculated for the four reports, adjusting by sequence case and country (*n*, adjusted mean, and 95% CI).

Analysis of opinion questionnaires was conducted by means of absolute and relative frequencies (*n*, %) for categorical variables and Likert-scale type variables, and central trend measures (*n*, mean, standard deviation [SD], median, Q1 and Q3, minimum and maximum, 95% CI, and mode) for continuous variables. The data analysis was adjusted by sequence case and country.

The data analysis for this article was generated using SAS/STAT software, Version 9.2 of the SAS System for Windows (Copyright (©) 2002–2008 by SAS Institute, Inc., Cary, NC).

## Results

### Participants

Thirty-seven participants answered the questionnaires and responded on the presence of the patterns; most of the participants were women (81.1%), endocrinologists (83.8%), and pediatricians (16.2%), with a mean time of 17 years of experience in diabetes and visiting a mean number of 18 CSII patients and 68 MDI patients.

### Physician opinion about the eDetecta module

**FIG. 2.**
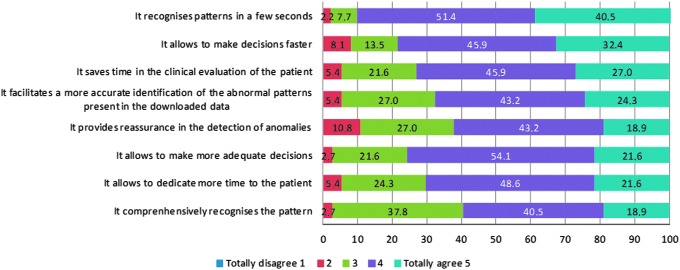
Physician opinion regarding the experience using the eDetecta module.

Regarding their experience using the eDetecta module, 91.9% of the clinicians agreed that eDetecta module recognizes the patterns in a few seconds, 78.3% concurred that it allows them to make decisions faster, and 72.9% agreed that it saves time in the clinical evaluation of the patient. 67.5% of the HCP agreed that the module facilitates a more accurate identification of the abnormal patterns present in the data downloaded, and 62.1% agreed that it provides reassurance regarding the detection of anomalies. 75.7% agreed that the module allows making more adequate decisions and 70.2% agreed that it allows to dedicate more time to the patient. Finally, 59.4% of the participants agreed that the module recognizes a comprehensive list of patterns (Fig. 2).

**FIG. 3.**
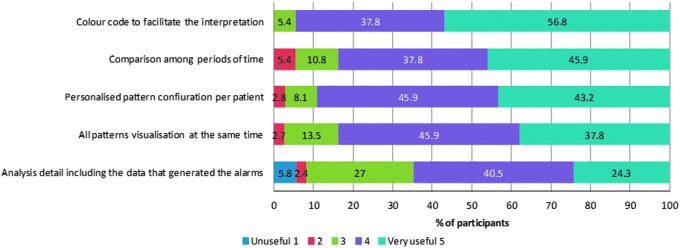
Physician perception of the usefulness of the eDetecta module.

Regarding the usefulness of the eDetecta module, physicians think that it is useful in automatic pattern detection. 94.6% agreed that its color code facilitates the interpretation of the data; 83.7% agreed that it is useful to compare between periods of time; 89.1% appreciate having a customizable configuration for each individual patient; 83.7% appreciate the visualization of all the patterns at the same time, and 64.8% appreciate the analysis detail, including the data that generated the alerts (Fig. 3).

97.3% of the participants agreed that, when changes in the configuration are needed, the available ranges cover the more frequent clinical situations. 83.7% of the participants agreed that the default configuration of the different patterns is applicable to most of the patients.

In their usual practice, 70.3% of the participating physicians kept the default configuration in all the patterns of the eDetecta module, while 29.7% changed some of them, mainly hypoglycemia (90.9%) and hyperglycemia (72.7%). Pregnancy was the main reason to change pattern configuration in all the patterns changed.

Participants were also asked about missed relevant patterns in the module and the answer was “no” for 89.2% of them.

### Analysis of the cases

To evaluate the eDetecta module, each investigator was asked to analyze the report corresponding to 4 cases; a maximum of 145 evaluations was obtained. CSII cases 1 and 2 were reviewed by 35 and 36 participants, respectively. All 37 endocrinologists reviewed the 2 cases under MDI therapy.

#### Time spent to analyze the cases

Mean time spent by physicians to analyze CSII cases was 17 min for case 1 (95% CI: 13.3–19.3) and 19 min for case 2 (95% CI: 15.6–22.2). For the MDI cases, mean time to complete case analysis was 12 min for case 1 (95% CI: 9.0–15.5) and 13 min for case 2 (95% CI: 9.3–15.8).

In contrast, the eDetecta module performed the pattern analysis automatically.

#### Global pattern detection

The comparison between the detection of the 19 patterns by HCP and the new eDetecta module showed a variable agreement depending on the case and existing patterns. Overall, the agreement was high (>65% of agreement in 16 patterns).

Table 2 provides the glycemic patterns defined by default in the eDetecta module.

**Table 2.** Glycemic Patterns Included in the Automated eDetecta Module

*Blocks*	*Patterns*	*Pattern definition*
Variability	Standard deviation	>70 mg/dL
	Low Blood Glucose Index	Moderate or high risk of severe hypoglycemia
	High Blood Glucose Index	Moderate or high risk of severe hyperglycemia
Hypoglycemia (glycemia <70 mg/dL)	Hypoglycemic trend	>1 Hypoglycemic event/day on 3 consecutive days
	Overcorrection hypoglycemia	>25% Hypoglycemic events preceded by a hyperglycemic event
	Hypoglycemia in a defined time block	Before/after breakfast, lunch, evening meal, bedtime, nocturnal
	Associated with a cartridge change^a^	Hypoglycemia present in >80% of cartridge changes
Hyperglycemia (>160 mg/dL)	Hyperglycemic trend	>2 Hyperglycemias/day for 7 consecutive days
	Associated with cartridge change delay^a^	Cartridge change delay of >1 day during which the >50% glycemic values are in the hyperglycemia range
	Overcorrection hyperglycemia	>25% of detected hyperglycemias (glycemia >200 mg/dL) are preceded by a hypoglycemia within <3 h
	Hyperglycemia in time block	Over 7 days, >3 hyperglycemias in the same time block (preprandial glycemia >150 mg/dL, postprandial glycemia >180 mg/dL)
	Missed bolus^a^	Over 4 weeks, >3 hyperglycemias related to missed bolus (intake of >20 g of CH unrelated to a hypoglycemia and without record of insulin bolus in the 2 h before the hyperglycemia)
Use of the system	Use of premarks and postmarks	<3 Glycemias/day with preprandial or postprandial marking on >50% of days
	Insulin and CH record	<3 Insulin and carbohydrate records per day
	Pump stopped^a^	>2 Stops a day on >50% of days
	Cartridge change (every 7 days)^a^	Cartridge change delay in >30% of changes
Treatment adherence	Frequency of BG tests	<4 Measurements/day on 80% of days
	BG test before bolus^a^	In >25% of boluses, no glycemia measurement in previous 30 min
	Bolus calculator use^a^	The bolus calculator was not used in >25% of the boluses

^a^ Continuous insulin infusion cases only.

BG, blood glucose.

Table 3 illustrates the patterns identified by the eDetecta module and level of agreement with the physician analysis (present/absent), both overall and for each individual pattern across the four cases.

**Table 3.** Level of Agreement Between Physician Identification (Present/Absent) of the Patterns Identified by the eDetecta Module

		*Percent physicians identifying presence/absence of a pattern in concordance with eDetecta (κ statistic)*				
*Blocks*	*Patterns*	*CSII case I*	*CSII case 2*	*MDI case I*	*MDI case 2*	*Global*
Variability	Standard deviation	100%, 0.99 (—)	100%, 0.99 (—)	83.8%, 0.67 (0.51, 0.84)	100%, 0.99 (—)	95.9%, 0.88 (0.77, 0.98)
	Low Blood Glucose Index	97.1%, 0.94 (0.86, 1.02)	86.1%, 0.72 (0.56, 0.88)	94.6%, 0.89 (0.79, 0.99)	81.1%, 0.62 (0.44, 0.80)	89.7%, 0.73 (0.60, 0.86)
	High Blood Glucose Index	91.4%, 0.83 (0.70, 0.96)	94.4%, 0.89 (0.78, 0.99)	94.6%, 0.89 (0.79, 0.99)	86.5%, 0.73 (0.57, 0.89)	91.7%, 0.62 (0.44, 0.81)
Hypoglycemia (<70 mg/dL)	Hypoglycemic trend	88.6%, 0.77 (0.62, 0.92)	63.9%, 0.28 (0.06, 0.50)	89.2%, 0.78 (0.64, 0.93)	**27.0%, −0.46 (−0.66, −0.26)**	66.9%, 0.36 (0.24, 0.48)
	Overcorrection hypoglycemia	91.4%, 0.83 (0.70, 0.96)	**33.3%, −0.33 (−0.56, −0.11)**	64.9%, 0.30 (0.08, 0.51)	64.9%, 0.30 (0.08, 0.51)	63.4%, 0.28 (0.13, 0.43)
	Hypoglycemia in a defined time block	74.3%, 0.48 (0.28, 0.69)	**55.6%, 0.11 (−0.12, 0.34)**	97.3%, 0.95 (0.87, 1.00)	86.5%, 0.73 (0.57, 0.89)	78.6%, 0.47 (0.32, 0.63)
	Associated with a cartridge change^a^	94.3%, 0.88 (0.78, 0.99)	100%, 0.99 (—)	—	—	97.2%, 0.94 (0.89, 0.99)
Hyperglycemia (>100 mg/dL)	Hyperglycemic trend	**2.9%, −0.94 (−1.02, −0.86)**	91.7%, 0.83 (0.71, 0.96)	70.3%, 0.41 (0.20, 0.61)	**10.8%, −0.78 (−0.93, −0.64)**	44.1%, 0.13 (0.06, 0.21)
	Cartridge change delay^a^	**60.0%, 0.20 (0.30, 0.43)**	77.8%, 0.56 (0.36, 0.75)	—	—	69.0%, 0.38 (0.27, 0.49)
	Overcorrection hyperglycemia	85.7%, 0.71 (0.55, 0.88)	**52.8%, 0.06 (−0.17, 0.29)**	73.0%, 0.46 (0.26, 0.66)	64.9%, 0.30 (0.08, 0.51)	69.0%, 0.38 (0.23, 0.53)
	Hyperglycemia in time block	97.1%, 0.94 (0.86, 1.02)	91.7%, 0.83 (0.71, 0.96)	**24.3%, −0.51 (−0.71, −0.32)**	86.5%, 0.73 (0.58, 0.89)	74.5%, 0.89 (0.81, 0.96)
	Missed bolus^a^	74.3%, 0.49 (0.28, 0.69)	86.1%, 0.72 (0.56, 0.89)	—	—	80.3%, 0.61 (0.48, 0.74)
Use of the system	Use of premarks and postmarks	82.9%, 0.66 (0.48, 0.83)	22.2%, **−**0.56 (**−**0.75, **−**0.36)	73.0%, 0.46 (0.26, 0.66)	81.1%, 0.62 (0.44, 0.80)	64.8%, 0.33 (0.18, 0.48)
	Insulin and carbs record	77.1%, 0.54 (0.34, 0.74)	66.7%, 0.33 (0.12, 0.55)	78.4%, 0.57 (0.38, 0.76)	89.2%, 0.78 (0.64, 0.93)	77.9%, 0.47 (0.32, 0.62)
	Pump stopped^a^	71.4%, 0.43 (0.22, 0.64)	61.1%, 0.22 (0.00, 0.45)	—	—	66.2%, 0.32 (0.17, 0.48)
	Cartridge change (every 7 days)^a^	57.1%, 0.14 (**−**0.09, 0.37)	75.0%, 0.50 (0.30, 0.70)	—	—	66.3%, 0.31 (0.12, 0.50)
Treatment adherence	Frequency of BG tests	71.4%, 0.43 (0.22, 0.64)	69.4%, 0.39 (0.18, 0.60)	59.5%, 0.19 (−0.03, 0.41)	70.3%, 0.41 (0.20, 0.61)	67.5%, 0.35 (0.24, 0.46)
	BG test before bolus^a^	71.4%, 0.43 (0.22, 0.64)	63.9%, 0.28 (0.06, 0.50)	—	—	67.6%, 0.37 (0.16, 0.57)
	Bolus calculator use^a^	82.9%, 0.66 (0.48, 0.83)	66.7%, 0.33 (0.12, 0.55)	—	—	74.6%, 0.51 (0.32, 0.70)

Shaded cells indicate a pattern is present for a given case, nonshaded cells indicate a pattern is not present. Data presented are the percentage of physicians correctly identifying the presence/absence of a given pattern and the associated Kappa statistic (95% CI). Bold numbers highlight those cases in which the level of agreement (κ) was ≤0.2 in hypoglycemia and hyperglycemia patterns. Level of agreement according to the Kappa Index (κ): No agreement (κ < 0), not significant (0 ≤ κ ≤ 0.20), medium (0.21 ≤ κ ≤ 0.4), moderate (0.41 ≤ κ ≤ 0.60), substantial (0.61 ≤ κ ≤ 0.80), almost perfect (κ > 0.80). Adjusted by sequence case and country.

^a^ CSII cases only.

CI, confidence interval; κ, Cohen's kappa coefficient.

#### Variability patterns

The overall level of agreement for the variability patterns was high. Presence/absence of the individual patterns was identified in agreement with eDetecta by 95.9% of physicians for SD, 89.7% for low blood glucose index, and 91.7% for high blood glucose index with κ values ranging from 0.62 to 0.88 (Table 2).

#### Hypoglycemia patterns

Across all four cases, identification of the presence/absence of “hypoglycemic trend,” “overcorrection hypoglycemia,” “hypoglycemia in a defined time block,” and “hypoglycemia associated with a cartridge change” was globally in agreement with eDetecta by 66.9%, 63.4%, 78.6%, and 97.2%, respectively.

Regarding “hypoglycemic trend” (see Table 1 for pattern definition), the highest level of agreement was achieved for the MDI case 1, where 89.2% of the participants identified the present pattern, and the lowest level of agreement was achieved in the MDI case 2, where the pattern was not present, but identified by 27.0% of the clinicians.

For “hypoglycemia due to overcorrection,” the highest level of agreement was obtained in the CSII case 1 (91.4%), where the pattern was not present, and the lowest level of agreement was obtained in the CSII case 2, where the pattern was present and identified by 33.3% of the clinicians.

In the pattern “hypoglycemia within a defined time block,” agreement ranged from 97.3% for the MDI case 1 to 55.6% for the CSII case 2, being the pattern present in both cases.

“Hypoglycemia associated with a cartridge change” was the pattern with the highest global agreement, reaching 94.3% in the CSII case 1 and 100% in the CSII case 2, being the pattern absent in both cases.

#### Hyperglycemia patterns

With regard to hyperglycemia patterns, global agreement was 44.1% for “hyperglycemic trend,” 69.0% for “cartridge change delay” and “overcorrection hyperglycemia,” 74.5% for “hyperglycemia in time block,” and 80.3% for “missed bolus.”

For “hyperglycemic trend,” the highest level of agreement was observed in the CSII case 2, where 91.7% of the participants recognized the present pattern, and the lowest level of agreement was observed in the CSII case 1, where the pattern was absent, but recognized only by 2.9% of the clinicians.

Regarding “hyperglycemia due to cartridge change delay,” the level of agreement was 60.0% for the CSII case 1 and 77.8% for the CSII case 2. In both cases, the pattern was not present.

For “hyperglycemia due to overcorrection,” the highest and lowest levels of agreement were observed in the CSII cases, ranging from 85.7% in the case 1 to 52.8% in the case 2, being the pattern absent in both cases.

For “hyperglycemia in time block,” the highest level of agreement was found in the CSII case 1, where the pattern was present and correctly identified by 97.1% of the clinicians, and the lowest level of agreement was observed in the MDI case 1, being the pattern present and identified by only 24.3% of the participants.

In the case of “hyperglycemia due to missed bolus,” the level of agreement was 74.3% for the CSII case 1 and 86.1% for the CSII case 2, being the pattern absent in both cases.

#### Use of system patterns

Global agreement for “use of system” patterns was moderate for the four patterns included. Regarding the individual patterns, the level of agreement was moderate-good in three of the four cases for the “preprandial and postprandial marks” pattern and in all four cases for the “insulin and carbohydrate [CH]” pattern, it was moderate in both CSII cases for “pump stopped,” and for “cartridge change,” it was poor in CSII case 1 and moderate in CSII case 2.

#### Treatment adherence patterns

Global agreement was moderate for all three treatment adherence patterns. Pattern “frequency of BG tests,” which was absent in all four cases, was identified in agreement with eDetecta by 71.4% of the physicians for CSII case 1, 69.4% of the physicians for CSII case 2, 59.5% of the physicians for MDI case 1, and 70.3% of the physicians for MDI case 2. Level of agreement was moderate-good for the patterns “BG test before bolus” and “bolus calculator use” with a global agreement of 67.3% and 74.6%, respectively, assessed for the two CSII cases.

### Physician opinion about data analysis

Regarding the frequency of use of data review methods, participating physicians suggested that data management tools are mainly used to review data in patients with insulin pumps (84.1%) and bolus calculators (79.2%). In contrast, these tools are only used in 39.6% of patients using a blood glucose meter, the manual review of patient logbooks being the most commonly used method.

When asked about the barriers found in using these tools, physicians highlighted time spent installing different software for different devices as the main difficulty.

They indicated that in clinical practice more time is needed to review insulin pump data (estimated mean time 20.5 min), followed by bolus calculator data (mean 14.7 min) and meter data (mean 11.5 min). In summary, physicians found the eDetecta module useful for automatic pattern detection. The majority of physicians (94.6%) indicated that it was a reliable tool. 83.8% of physicians were generally satisfied with the module and 89.2% of physicians would recommend emminens eConecta with the eDetecta module to their colleagues.

## Discussion

In this case- and survey-based evaluation of glycemic pattern recognition with the eDetecta module, good level of agreement was shown between physicians and eDetecta pattern recognition, and physicians reported this tool to be useful, reliable, and comprehensive for clinical practice. Previous studies have demonstrated the utility of pattern detection software in achieving more rapid and accurate pattern detection compared with nonautomated review of clinical data.^6,7^

Good level of agreement was shown between physician and eDetecta pattern recognition for patterns relating to glycemic variability. In these cases, the eDetecta module could prevent time spent by physicians on mental calculations to identify clinically relevant patterns. The module can detect automatically moderate or high ranges of Low Blood Glucose Index and High Blood Glucose Index, and thresholds for SD and percentage coefficient of variation for glucose (% CV, obtained from the calculation: [SD of glucose/mean glucose] × 100) can be configured. Having a glycemic variability pattern related to % CV can facilitate an easy identification of stable or unstable glycemic status. In a recent study, a relationship between the % CV and frequency of hypoglycemia was found, which was significantly greater in subjects who had a CV value >36% than in those who were below this threshold.^8^ In the other patterns (hypoglycemia, hyperglycemia, use of the system, and treatment adherence), low level of agreement was observed in at least one of the cases, but low level of agreement in the same pattern was never present for all four cases.

Patterns of particular clinical relevance where the level of agreement between the eDetecta pattern identification and clinical opinion was low included the following: “hypoglycemic/hyperglycemic trends,” “overcorrection-associated hypoglycemia/hyperglycemia,” and identification of “hypoglycemic/hyperglycemic patterns within defined time blocks.” A deeper analysis focusing on the results with low level of agreement was conducted.

One of the main causes of low level of agreement was the understanding of the provided definition for every pattern. For example, “Hypoglycemic Trend” was initially defined as “more than 1 hypoglycemia per day over 3 consecutive days.” This definition led to a low level of agreement (κ = −0.46) in MDI case 2, where only 27% of participants considered that the trend was present. According to participants, “Hypoglycemia Trend” definition would be more clinically relevant if “>1” was changed to “at least 1 or more hypoglycemias per day over 3 consecutive days,” potentially improving agreement. Similarly, when “Hyperglycemic trend” is defined as “more than 2 hyperglycemias a day for 7 consecutive days,” a low level of agreement is obtained in MDI case 2 (κ = −0.78); the authors suggested to change “>2” for “2 or more hyperglycemias a day for 4 consecutive days,” possibly increasing agreement, since this would have clinical sense.

Pattern definition was reached by consensus of a scientific committee^3^ composed by a group of endocrinologists and pediatric experts in diabetes, with the aim to cover most patients and signal those situations that need attention according to clinical experience; however, the results showed that some of the default pattern configurations do not fit with the usual clinical practice. That is why it is important to highlight that eDetecta pattern definitions are customizable to adjust the proper values for every patient or group of patients by the HCPs.

Low level of agreement was also present in patterns where definitions involved a rate like “overcorrection hypoglycemia and hyperglycemia” (Table 2). Regarding “hypoglycemia by hyperglycemia overcorrection,” the low level of agreement could be explained due to the difficulty in mentally calculating the 25% of cases preceded by a hyperglycemic event, as described in the pattern definition. The same pattern in MDI case 2 reached a higher percentage of identification because the ratio between the hypoglycemias and previous hyperglycemias was more evident.

When analyzing the low level of agreement in the CSII case 2 for “hyperglycemia by hypoglycemia overcorrection,” the proposed definition did not completely match the perception of the clinicians. According to the authors, the definition would be more clinically relevant if the denominator of the equation is not the total number of hyperglycemias and the threshold (glycemia >200 mg/dL) is higher, potentially improving agreement.

Finally, low level of agreement was also present in cases where contrasting patterns were present. For example, in MDI case 1, the level of agreement in the pattern “Hyperglycemia in Time Block” was low (κ = −0.51). In this report, there was a predominant hypoglycemic context at the same time. For physicians, “Hyperglycemia in Time Block” was not relevant in the presence of “Hypoglycemia in Time Block” and therefore, the level of agreement with eDetecta was low. However, eDetecta did actually signal the presence of both patterns.

The use of this type of pattern detection software can also provide caregivers further information on the use of devices, which is closely related to therapy adherence and is difficult to obtain otherwise. Patterns related to the frequency of blood glucose tests before bolus and the use of bolus calculators had a good level of agreement and were appreciated by the participants. Future studies will show if the automatic tool eDetecta has a clinical impact in diabetes management.

This evaluation was conducted in a version only for professional use. The future availability of a version for patient use could help the empowerment of the patient, and probably improve the knowledge of the disease and promote self-care behaviors in their daily life.^9^

The results of this research have demonstrated the clinical utility and acceptability of the eDetecta module to support physicians in evaluating real-life glucose downloads, both in terms of time invested and pattern identification. This module is already in use in different centers in Spain and Portugal; more ranges in pattern definition will be configured in the module to include as many patients as possible in the default values, although the capability to adjust the alerts to the needs of every patient or group of patients by the clinicians will remain as one of the features of the module, allowing a personalized management of the patient with diabetes.

## Supplementary Material

Supplemental data
