# Supplementary material for: Evaluation of a New Digital Automated Glycemic Pattern Detection Tool
Source: Diabetes Technol Ther. 2017 Nov 1;19(11):633–40. doi: 10.1089/dia.2017.0180 (PMC5689116; doi:10.1089/dia.2017.0180)
Supplement: Supplemental data [file Supp_Data.pdf]

Day

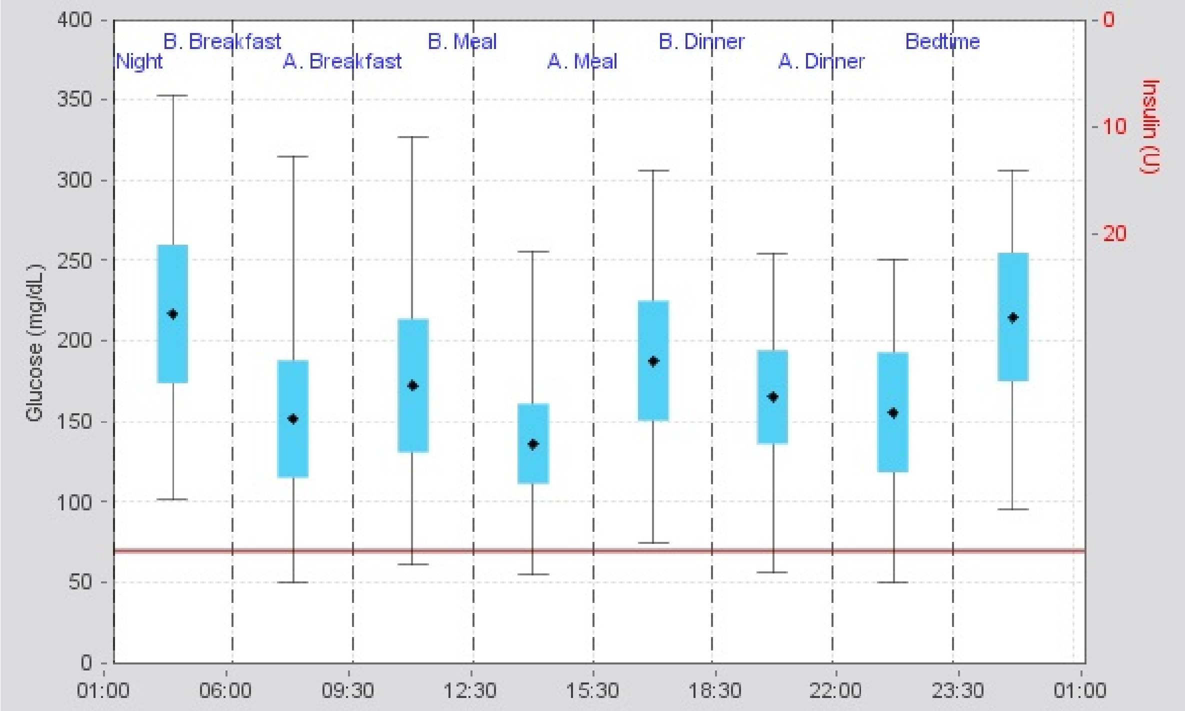
Night
B. Breakfast
A. Breakfast
B. Meal
A. Meal
B. Dinner
A. Dinner
Bedtime
Glucose (mg/dL)
Insulin (U)
0
50
100
150
200
250
300
350
400
0
10
20
01:00
06:00
09:30
12:30
15:30
18:30
22:00
23:30
01:00

Week

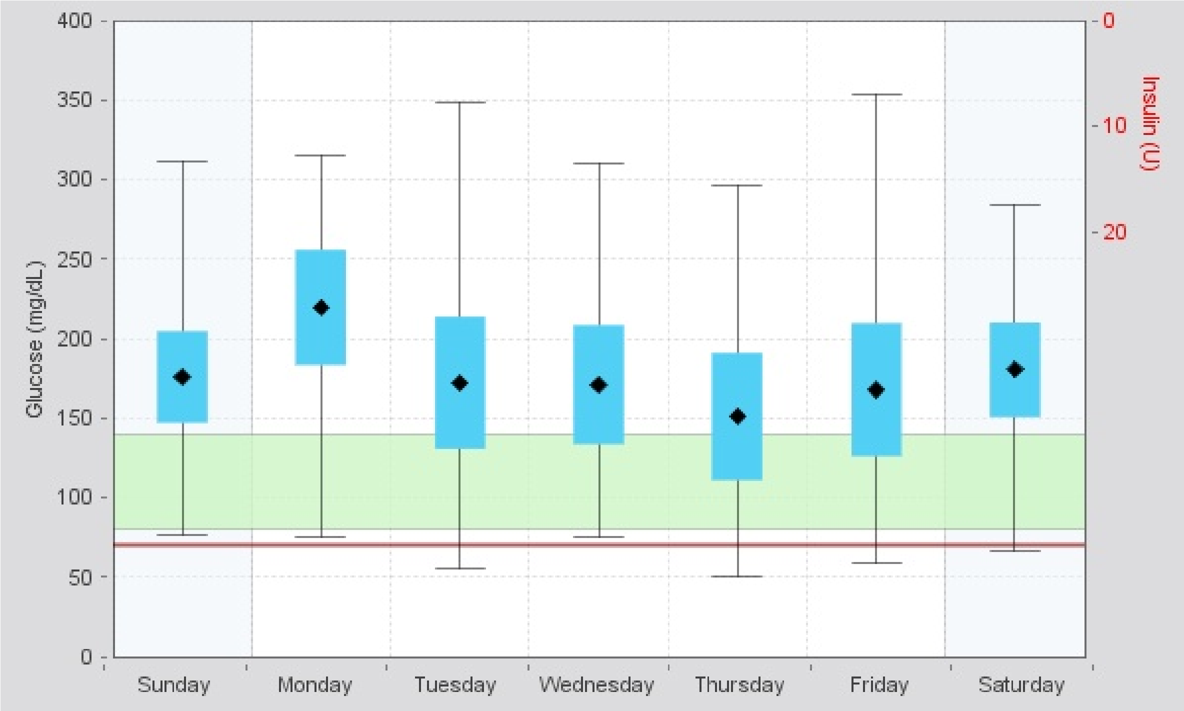
Glucose (mg/dL)
Insulin (U)
0
50
100
150
200
250
300
350
400
0
10
20
Sunday
Monday
Tuesday
Wednesday
Thursday
Friday
Saturday

Daily Trend

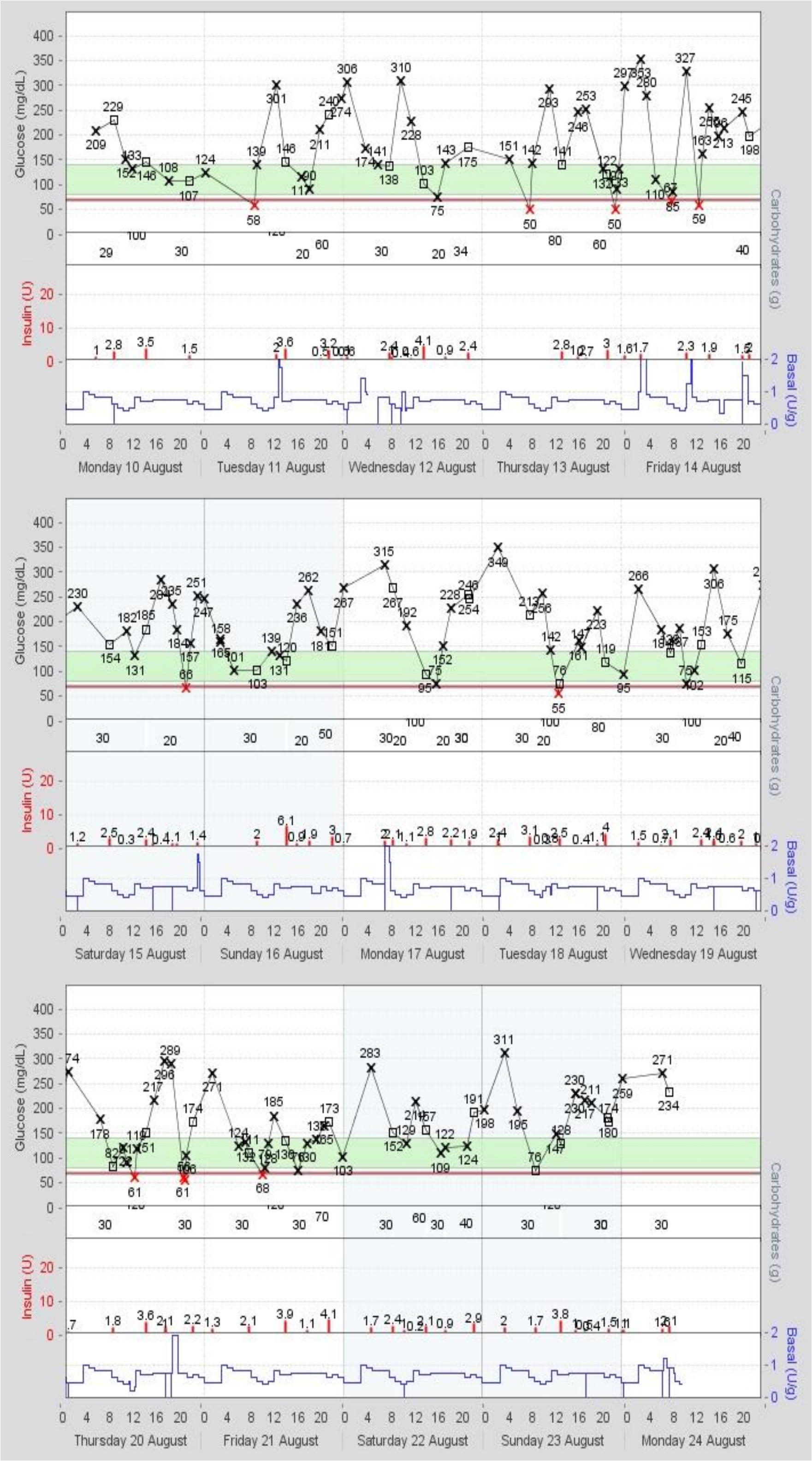
Glucose (mg/dL)
Insulin (U)
Carbohydrates (g)
Basal (U/g)
Monday 10 August
Tuesday 11 August
Wednesday 12 August
Thursday 13 August
Friday 14 August
Saturday 15 August
Sunday 16 August
Monday 17 August
Tuesday 18 August
Wednesday 19 August
Thursday 20 August
Friday 21 August
Saturday 22 August
Sunday 23 August
Monday 24 August

Trend

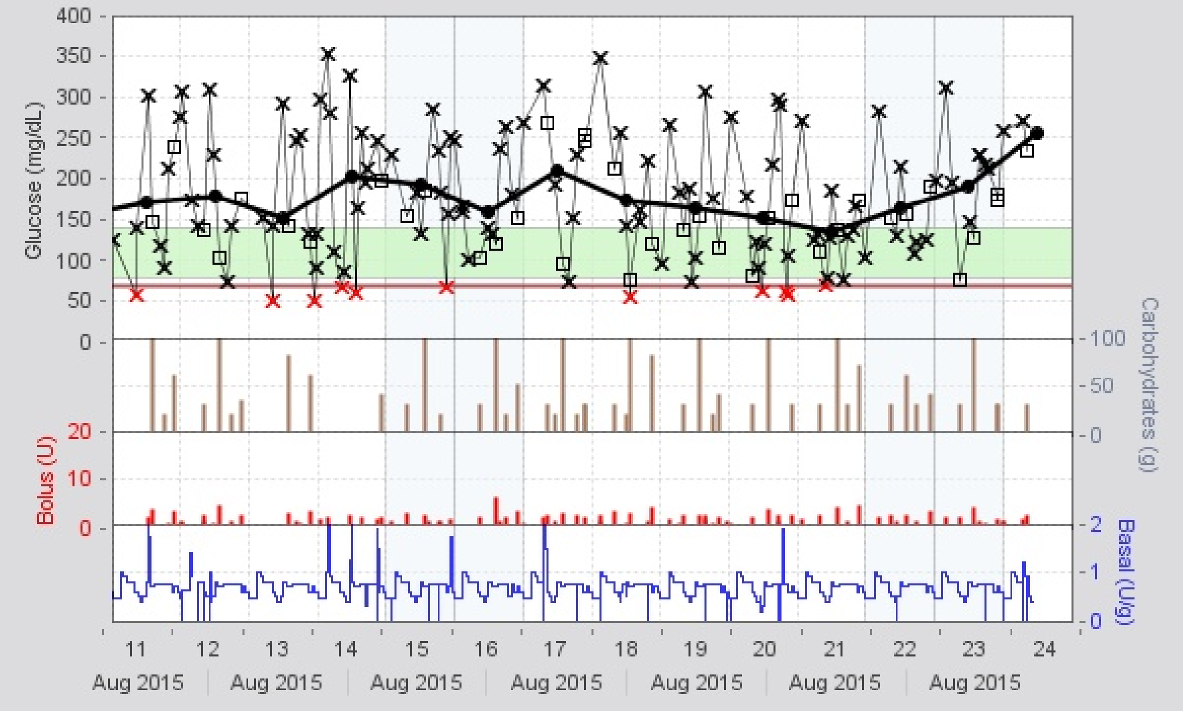
Glucose (mg/dL)
400
350
300
250
200
150
100
50
0
Carbohydrates (g)
100
50
0
Bolus (U)
20
10
0
Basal (U/g)
2
1
0
11 Aug 2015
12 Aug 2015
13 Aug 2015
14 Aug 2015
15 Aug 2015
16 Aug 2015
17 Aug 2015
18 Aug 2015
19 Aug 2015
20 Aug 2015
21 Aug 2015
22 Aug 2015
23 Aug 2015
24

TIME INTERVAL I FROM 11/08/15 TO 24/08/15

PUMP STATISTICS

NUMBER OF TESTS	BG TEST FREQUENCY	MBG TOTAL	MBG BEFORE MEAL	MBG AFTER MEAL
154	11 (11)	173 mg/dL	156 mg/dL	0 mg/dL

LOWEST VALUE	TARGET RANGE %		HYPOS	SD	HBGI	LBGI
50 mg/dL	ABOVE	61%	11	74 mg/dL	10.1	1.4
HIGHEST VALUE	WITHIN	27%	HYPO LIMIT			
353 mg/dL	UNDER	12%	70 mg/dL			

INSULIN BOLUS

EVALUATED	FREQUENCY/ DAY	% BOLUS TYPE				% BOLUS RECOMMEN	MEAN UI/DAY	LOWEST VALUE	HIGHEST VALUE
92	6.6	92	0	0	8	90.2	11.6	0.2	6.1

INTAKES

EVALUATED RESULTS	FREQUENCY/DAY	MEAN CH/DAY	LOWEST VALUE CH	HIGHEST VALUE CH
47	3.4	183.9	20	200

BASAL/BOLUS

	16.7 U/Day	58%
	12.1 U/Day	42%
+	28.8 U/Day	

BASAL

25	4	0	0	1%

BASAL/BOLUS

PRIME INFUSION SET	CARTRIDGE CHANGED
0.5	3

Legend/Caption

Glycemic Variability. Risk Indexes

Risk class	LBGI	HBGI
Minimal Risk	<=1,1	<=5
Low Risk	1,1-2,5	5-10
Moderate Risk	2,5-5	10-15
High Risk	>5	>15

Kovatchev, Diabetes Care 1998

Comb. Record List

Day	Date	Time	Blood Glucose mg/dL			Insulin 1	Insulin 2	Insulin 3		Insulin Pump	Comments
Tuesday	11/08/2015	00:00				9.43			1		26.51
Tuesday	11/08/2015	00:07	124						1		
Tuesday	11/08/2015	03:00							1		
Tuesday	11/08/2015	04:00							1		
Tuesday	11/08/2015	05:00							1		
Tuesday	11/08/2015	08:00							1		
Tuesday	11/08/2015	08:38	58	H					1		
Tuesday	11/08/2015	08:59	139						1		
Tuesday	11/08/2015	09:00							1		
Tuesday	11/08/2015	10:00							1		
Tuesday	11/08/2015	11:00							1		
Tuesday	11/08/2015	12:00							1		
Tuesday	11/08/2015	12:27	301			2.0			1		
Tuesday	11/08/2015	12:43								250% IU	Stop<br>Start-up<br>dur 00:45 h<br>Prime Insulin Pump
Tuesday	11/08/2015	13:00							1	250%	
Tuesday	11/08/2015	13:28							1		End of TBR

Legend/Caption

Hypoglycaemia	Hypoglycaemia symptoms	Before meal	After meal	Asterisk
Before sport (23)	During sport (45)	After sport (24)	Stress (29)	Disease (31)

Day	Date	Time	Blood Glucose mg/dL	Blood Glucose symbols	Carbohydrates	Insulin 1 / 2 / 3	Basal	Insulin Pump	Comments
Tuesday	11/08/2015	14:00	146	□	120		1		
Tuesday	11/08/2015	14:01				3.6	1		
Tuesday	11/08/2015	15:00					1		
Tuesday	11/08/2015	16:45	117				1		
Tuesday	11/08/2015	17:36					1	W1	Cartridge low
Tuesday	11/08/2015	18:09	90		20		1		Other Exercise 1
Tuesday	11/08/2015	20:00	211				1		
Tuesday	11/08/2015	20:01				0.5	1		
Tuesday	11/08/2015	21:00					1		
Tuesday	11/08/2015	21:35	240	□	60	3.2 1.10 / 2.10 3:07 h	1		
Tuesday	11/08/2015	22:00					1		
Tuesday	11/08/2015	23:00					1		
Tuesday	11/08/2015	23:34	274			0.6	1		
Wednesday	12/08/2015	00:00				13.8 7	1		29.56
Wednesday	12/08/2015	00:42	306			1.0		W8 ■	Stop Bolus cancelled
Wednesday	12/08/2015	00:43				0.6	1		Start-up
Wednesday	12/08/2015	00:53					1	140%	dur 02:51 h

Legend/Caption

Hypoglycaemia	Hypoglycaemia symptoms	Before meal	After meal	Asterisk
Before sport (23)	During sport (45)	After sport (24)	Stress (29)	Disease (31)

Day	Date	Time	Blood Glucose mg/dL	Blood Glucose ✱ ⊖ □ ◿ ⚡ ∿	🍴	Insulin 1 2 3	📊	Insulin Pump	Comments
Wednesday	12/08/2015	03:00					1	140%	
Wednesday	12/08/2015	03:44	174				1		End of TBR (cancelled)
Wednesday	12/08/2015	04:00					1		
Wednesday	12/08/2015	04:12						■ E4	Stop Occlusion
Wednesday	12/08/2015	05:53					1	▶	Start-up
Wednesday	12/08/2015	05:55	141				1		
Wednesday	12/08/2015	07:55	138	□	30		1		
Wednesday	12/08/2015	07:56				2.4 ⊥☀	1		
Wednesday	12/08/2015	08:00					1		
Wednesday	12/08/2015	08:09						■	Stop
Wednesday	12/08/2015	08:10					1		Cartridge changed
Wednesday	12/08/2015	08:17					1	25.00 IU	Prime Insulin Pump
Wednesday	12/08/2015	08:18					1	▶ 15.80 IU	Start-up<br>Prime Insulin Pump
Wednesday	12/08/2015	08:19				1.0 ⊥	1		
Wednesday	12/08/2015	09:00					1		
Wednesday	12/08/2015	09:56	310			0.4 ⊥☀		■	Stop
Wednesday	12/08/2015	09:57					1	▶ 9.10 IU	Start-up<br>Prime Insulin Pump

Legend/Caption

⊖ Hypoglycaemia	○ Hypoglycaemia symptoms	□ Before meal	■ After meal	✱ Asterisk
◿ Before sport (23)	◿ During sport (45)	◿ After sport (24)	⚡ Stress (29)	∿ Disease (31)

Day	Date	Time	Blood Glucose mg/dL	Blood Glucose markers	Meal	Insulin 1	Insulin 2	Insulin 3	Basal	Insulin Pump	Comments
Wednesday	12/08/2015	10:00							1		
Wednesday	12/08/2015	10:11							1	250%	dur 00:30 h
Wednesday	12/08/2015	10:41							1		End of TBR
Wednesday	12/08/2015	11:00							1		
Wednesday	12/08/2015	11:43	228			0.6			1		
Wednesday	12/08/2015	12:00							1		
Wednesday	12/08/2015	13:00							1		
Wednesday	12/08/2015	13:56	103	Before meal	140				1		
Wednesday	12/08/2015	13:57				4.1			1		
Wednesday	12/08/2015	15:00							1		
Wednesday	12/08/2015	16:20	75						1		
Wednesday	12/08/2015	17:39	143		20	0.9			1		
Wednesday	12/08/2015	21:00							1		
Wednesday	12/08/2015	21:33	175	Before meal	34				1		
Wednesday	12/08/2015	21:34				2.4			1		
Wednesday	12/08/2015	22:00							1		
Wednesday	12/08/2015	23:00							1		

Legend/Caption

Hypoglycaemia	Hypoglycaemia symptoms	Before meal	After meal	Asterisk
Before sport (23)	During sport (45)	After sport (24)	Stress (29)	Disease (31)

Day	Date	Time	Blood Glucose mg/dL	Blood Glucose markers	Meal	Insulin 1	Insulin 2	Insulin 3	Basal	Insulin Pump	Comments
Thursday	13/08/2015	00:00				7.7			1		23.95
Thursday	13/08/2015	03:00							1		
Thursday	13/08/2015	04:00							1		
Thursday	13/08/2015	04:35	151						1		
Thursday	13/08/2015	05:00							1		
Thursday	13/08/2015	08:00							1		
Thursday	13/08/2015	08:16	50	H					1		
Thursday	13/08/2015	08:35	142						1		
Thursday	13/08/2015	09:00							1		
Thursday	13/08/2015	10:00							1		
Thursday	13/08/2015	11:00							1		
Thursday	13/08/2015	11:34	293						1		
Thursday	13/08/2015	12:00							1		
Thursday	13/08/2015	13:00							1		
Thursday	13/08/2015	13:48	141	□	80				1		
Thursday	13/08/2015	13:49				2.8	0.80 / 2.00 3:59 h		1		
Thursday	13/08/2015	15:00							1		

Legend/Caption

Hypoglycaemia	Hypoglycaemia symptoms	Before meal	After meal	Asterisk
Before sport (23)	During sport (45)	After sport (24)	Stress (29)	Disease (31)

Day	Date	Time	Blood Glucose mg/dL	Blood Glucose symbols	Meal	Insulin 1	Insulin 2	Insulin 3	Basal	Insulin Pump	Comments
Thursday	13/08/2015	16:35	246						1		
Thursday	13/08/2015	16:36				1.2			1		
Thursday	13/08/2015	17:59	253			0.7			1		
Thursday	13/08/2015	20:48	132						1		
Thursday	13/08/2015	21:00							1		
Thursday	13/08/2015	21:33	122	□	60				1		
Thursday	13/08/2015	21:34				3.0			1		
Thursday	13/08/2015	22:00							1		
Thursday	13/08/2015	22:58	50	H					1		
Thursday	13/08/2015	23:00							1		
Thursday	13/08/2015	23:17	91						1		
Thursday	13/08/2015	23:32	133						1		
Friday	14/08/2015	00:00				11.0			1		30.09 +
Friday	14/08/2015	00:36	297			1.6			1		
Friday	14/08/2015	03:00							1		
Friday	14/08/2015	03:27	353			1.7			1	250%	dur 00:30 h
Friday	14/08/2015	03:57							1	250%	dur 00:30 h<br>End of TBR

Legend/Caption

Hypoglycaemia	Hypoglycaemia symptoms	Before meal	After meal	Asterisk
Before sport (23)	During sport (45)	After sport (24)	Stress (29)	Disease (31)

Day	Date	Time	Blood Glucose mg/dL			Insulin 1	2	3		Insulin Pump	Comments
Friday	14/08/2015	04:00							1	250%	
Friday	14/08/2015	04:26	280						1		
Friday	14/08/2015	04:27							1		End of TBR
Friday	14/08/2015	05:00							1		
Friday	14/08/2015	05:53	110						1		
Friday	14/08/2015	08:00							1		
Friday	14/08/2015	08:41	67	H					1		
Friday	14/08/2015	08:59	85						1		
Friday	14/08/2015	09:00							1		
Friday	14/08/2015	10:00							1		
Friday	14/08/2015	11:00							1		
Friday	14/08/2015	11:11	327			2.3			1		
Friday	14/08/2015	11:12							1	250%	dur 01:00 h
Friday	14/08/2015	12:00							1	250%	
Friday	14/08/2015	12:12							1		End of TBR
Friday	14/08/2015	13:00							1		
Friday	14/08/2015	13:27	59	H					1		

Legend/Caption

Hypoglycaemia	Hypoglycaemia symptoms	Before meal	After meal	Asterisk
Before sport (23)	During sport (45)	After sport (24)	Stress (29)	Disease (31)

Day	Date	Time	Blood Glucose mg/dL	Blood Glucose markers	Meal	Insulin 1	Insulin 2	Insulin 3	Profile	Insulin Pump	Comments
Friday	14/08/2015	13:59	163						1		
Friday	14/08/2015	15:00							1		
Friday	14/08/2015	15:14	255			1.9			1		
Friday	14/08/2015	16:43	196						1		
Friday	14/08/2015	16:59							1	40%	dur 00:43 h
Friday	14/08/2015	17:42	213						1		End of TBR (cancelled)
Friday	14/08/2015	20:52	245			1.5			1		
Friday	14/08/2015	20:53									Stop
Friday	14/08/2015	20:55							1	20.10 IU	Start-up<br>Prime Insulin Pump
Friday	14/08/2015	20:56							1	250%	dur 00:30 h
Friday	14/08/2015	21:00							1	250%	
Friday	14/08/2015	21:26							1	250%	dur 00:30 h<br>End of TBR
Friday	14/08/2015	21:56							1		End of TBR
Friday	14/08/2015	22:00							1		
Friday	14/08/2015	22:08	198	□	40				1		
Friday	14/08/2015	22:09				2.0			1		
Friday	14/08/2015	23:00							1		

Legend/Caption

Hypoglycaemia	Hypoglycaemia symptoms	Before meal	After meal	Asterisk
Before sport (23)	During sport (45)	After sport (24)	Stress (29)	Disease (31)

Day	Date	Time	Blood Glucose mg/dL	✱ □		Insulin 1	2	3		Insulin Pump	Comments
Saturday	15/08/2015	00:00				11.3			1		27.94
Saturday	15/08/2015	02:05	230			1.2			1		
Saturday	15/08/2015	02:08									Stop
Saturday	15/08/2015	02:09							1	13.40 IU	Start-up<br>Prime Insulin Pump
Saturday	15/08/2015	03:00							1		
Saturday	15/08/2015	04:00							1		
Saturday	15/08/2015	05:00							1		
Saturday	15/08/2015	05:09							1	W1	Cartridge low
Saturday	15/08/2015	07:39	154	□	30				1		
Saturday	15/08/2015	07:40				2.5			1		
Saturday	15/08/2015	08:00							1		
Saturday	15/08/2015	09:00							1		
Saturday	15/08/2015	10:00							1		
Saturday	15/08/2015	10:37	182			0.3			1		
Saturday	15/08/2015	11:00							1		
Saturday	15/08/2015	11:58	131						1		
Saturday	15/08/2015	12:00							1		

Legend/Caption

Hypoglycaemia	Hypoglycaemia symptoms	Before meal	After meal	Asterisk
Before sport (23)	During sport (45)	After sport (24)	Stress (29)	Disease (31)

Day	Date	Time	Blood Glucose mg/dL		Carbs	Insulin 1	Insulin 2	Insulin 3	Basal	Insulin Pump	Comments
Saturday	15/08/2015	13:00							1		
Saturday	15/08/2015	13:50	185	□	140	2.4	1.50 / 0.90 1:06 h		1		
Saturday	15/08/2015	14:56								W8	Stop<br>Bolus cancelled
Saturday	15/08/2015	15:00							1	25.00 IU	Prime Insulin Pump
Saturday	15/08/2015	15:01							1	8.90 IU	Start-up<br>Prime Insulin Pump
Saturday	15/08/2015	15:02				1.0			1	W8	Bolus cancelled
Saturday	15/08/2015	16:25	284			0.4			1		
Saturday	15/08/2015	18:26	235			1.1					Stop
Saturday	15/08/2015	18:28							1	10.80 IU	Start-up<br>Prime Insulin Pump
Saturday	15/08/2015	19:19	184		20	1.0			1		
Saturday	15/08/2015	20:54	66	H					1		
Saturday	15/08/2015	21:00							1		
Saturday	15/08/2015	21:35	157						1		
Saturday	15/08/2015	22:00							1		
Saturday	15/08/2015	22:44	251			1.4			1		
Saturday	15/08/2015	22:46							1	250%	dur 00:30 h
Saturday	15/08/2015	23:00							1	250%	

Legend/Caption

Hypoglycaemia	Hypoglycaemia symptoms	Before meal	After meal	Asterisk
Before sport (23)	During sport (45)	After sport (24)	Stress (29)	Disease (31)

Day	Date	Time	Blood Glucose mg/dL	Blood Glucose markers	Meal	Insulin 1	Insulin 2	Insulin 3	Basal	Insulin Pump	Comments
Saturday	15/08/2015	23:16							1		End of TBR
Saturday	15/08/2015	23:56	247						1		
Saturday	15/08/2015	23:57								4.00 IU	Stop<br>Start-up<br>Prime Insulin Pump
Sunday	16/08/2015	00:00				13.9			1		29.76
Sunday	16/08/2015	02:49	158						1		
Sunday	16/08/2015	02:50	165						1		
Sunday	16/08/2015	03:00							1		
Sunday	16/08/2015	04:00							1		
Sunday	16/08/2015	05:00							1		
Sunday	16/08/2015	05:08	101						1		
Sunday	16/08/2015	08:00							1		
Sunday	16/08/2015	09:00							1		
Sunday	16/08/2015	09:06	103	□ (Before meal)	30	2.0			1		
Sunday	16/08/2015	10:00							1		
Sunday	16/08/2015	11:00							1		
Sunday	16/08/2015	11:37	139						1		
Sunday	16/08/2015	12:00							1		

Legend/Caption

Hypoglycaemia	Hypoglycaemia symptoms	Before meal	After meal	Asterisk
Before sport (23)	During sport (45)	After sport (24)	Stress (29)	Disease (31)

Day	Date	Time	Blood Glucose mg/dL	Blood Glucose markers	Carbs	Insulin 1	Insulin 2	Insulin 3	Basal	Insulin Pump	Comments
Sunday	16/08/2015	12:59	131						1		
Sunday	16/08/2015	13:00							1		
Sunday	16/08/2015	14:13	120	Before meal	200				1		
Sunday	16/08/2015	14:14				6.1	1.80 / 4.30 3:58 h		1		
Sunday	16/08/2015	15:00							1		
Sunday	16/08/2015	16:03	236			0.9			1		
Sunday	16/08/2015	18:02	262		20				1		
Sunday	16/08/2015	18:03				1.9			1		
Sunday	16/08/2015	18:27							1	70%	dur 01:45 h
Sunday	16/08/2015	20:03	181						1		
Sunday	16/08/2015	20:12							1		End of TBR
Sunday	16/08/2015	21:00							1		
Sunday	16/08/2015	22:00							1		
Sunday	16/08/2015	22:07	151	Before meal	50	3.0			1		
Sunday	16/08/2015	23:00							1		
Monday	17/08/2015	00:00				12.8			1		30.16 +
Monday	17/08/2015	00:05	267			0.7			1		

Legend/Caption

Hypoglycaemia	Hypoglycaemia symptoms	Before meal	After meal	Asterisk
Before sport (23)	During sport (45)	After sport (24)	Stress (29)	Disease (31)

Day	Date	Time	Blood Glucose mg/dL	Blood Glucose symbols	Meal	Insulin 1	Insulin 2	Insulin 3	Basal	Insulin Pump	Comments
Monday	17/08/2015	03:00							1		
Monday	17/08/2015	04:00							1		
Monday	17/08/2015	05:00							1		
Monday	17/08/2015	07:08	315			2.0				■	Stop
Monday	17/08/2015	07:10							1	250% ▶<br>18.40 IU	Start-up<br>dur 00:30 h<br>Prime Insulin Pump
Monday	17/08/2015	07:40							1	250%	dur 00:30 h<br>End of TBR
Monday	17/08/2015	08:00							1	250%	
Monday	17/08/2015	08:10							1		End of TBR
Monday	17/08/2015	08:37	267	□	30	2.1			1		
Monday	17/08/2015	09:00							1		
Monday	17/08/2015	10:00							1		
Monday	17/08/2015	10:57	192		20				1		
Monday	17/08/2015	10:58				1.1			1		
Monday	17/08/2015	11:00							1		
Monday	17/08/2015	12:00							1		
Monday	17/08/2015	13:00							1		
Monday	17/08/2015	14:16	95	□	100				1		

Legend/Caption

Hypoglycaemia	Hypoglycaemia symptoms	Before meal	After meal	Asterisk
Before sport (23)	During sport (45)	After sport (24)	Stress (29)	Disease (31)

Day	Date	Time	Blood Glucose mg/dL	Blood Glucose markers	Carbohydrates	Insulin 1	Insulin 2	Insulin 3	Basal	Insulin Pump	Comments
Monday	17/08/2015	14:17				2.8			1		
Monday	17/08/2015	15:00							1		
Monday	17/08/2015	16:01	75						1		
Monday	17/08/2015	17:09	152						1		
Monday	17/08/2015	18:32	228		20				1		
Monday	17/08/2015	18:33				2.2				■	Stop
Monday	17/08/2015	18:34							1	▶ 3.40 IU	Start-up Prime Insulin Pump
Monday	17/08/2015	21:00							1		
Monday	17/08/2015	21:40	254	□	30				1		
Monday	17/08/2015	21:42	246	□	30	1.9			1		
Monday	17/08/2015	22:00							1		
Monday	17/08/2015	23:00							1		
Tuesday	18/08/2015	00:00				15.6			1		32.16
Tuesday	18/08/2015	02:46	349			2.4			1		
Tuesday	18/08/2015	02:49								■	Stop
Tuesday	18/08/2015	02:50							1	▶ 8.40 IU	Start-up Prime Insulin Pump
Tuesday	18/08/2015	02:53				1.0			1		

Legend/Caption

Hypoglycaemia	Hypoglycaemia symptoms	Before meal	After meal	Asterisk
Before sport (23)	During sport (45)	After sport (24)	Stress (29)	Disease (31)

			Blood Glucose			Insulin				Insulin Pump	
Day	Date	Time	mg/dL			1	2	3			Comments
Tuesday	18/08/2015	03:00							1		
Tuesday	18/08/2015	04:00							1		
Tuesday	18/08/2015	05:00							1		
Tuesday	18/08/2015	08:00							1		
Tuesday	18/08/2015	08:10	213	□	30				1		
Tuesday	18/08/2015	08:11				3.1			1		
Tuesday	18/08/2015	09:00							1		
Tuesday	18/08/2015	10:00							1		
Tuesday	18/08/2015	10:20	256			0.3			1		
Tuesday	18/08/2015	10:22							1	150%	dur 01:30 h
Tuesday	18/08/2015	11:00							1	150%	
Tuesday	18/08/2015	11:51	142		20				1		
Tuesday	18/08/2015	11:52				0.8			1		End of TBR (cancelled)
Tuesday	18/08/2015	12:00							1		
Tuesday	18/08/2015	13:00							1		
Tuesday	18/08/2015	13:12	55	H					1		
Tuesday	18/08/2015	13:26	76	□	100				1		

Legend/Caption

Hypoglycaemia	Hypoglycaemia symptoms	Before meal	After meal	Asterisk
Before sport (23)	During sport (45)	After sport (24)	Stress (29)	Disease (31)

Day	Date	Time	Blood Glucose mg/dL	Blood Glucose markers	Meal	Insulin 1	Insulin 2	Insulin 3	Basal	Insulin Pump	Comments
Tuesday	18/08/2015	13:27				2.5			1		
Tuesday	18/08/2015	15:00							1		
Tuesday	18/08/2015	16:40	161						1		
Tuesday	18/08/2015	17:05	147						1		
Tuesday	18/08/2015	17:06				0.4			1		
Tuesday	18/08/2015	19:51	223			1.1				Stop	Stop
Tuesday	18/08/2015	19:53							1	Start-up 14.50 IU	Start-up Prime Insulin Pump
Tuesday	18/08/2015	21:00							1		
Tuesday	18/08/2015	21:16	119	Before meal	80	4.0			1		
Tuesday	18/08/2015	22:00							1		
Tuesday	18/08/2015	23:00							1		
Wednesday	19/08/2015	00:00				14.3			1		30.42
Wednesday	19/08/2015	00:29	95						1		
Wednesday	19/08/2015	02:59	266			1.5			1		
Wednesday	19/08/2015	03:00							1		
Wednesday	19/08/2015	04:00							1		
Wednesday	19/08/2015	05:00							1		

Legend/Caption

Hypoglycaemia	Hypoglycaemia symptoms	Before meal	After meal	Asterisk
Before sport (23)	During sport (45)	After sport (24)	Stress (29)	Disease (31)

Day	Date	Time	Blood Glucose mg/dL	Blood Glucose markers	Carbohydrates	Insulin 1	Insulin 2	Insulin 3	Basal	Insulin Pump	Comments
Wednesday	19/08/2015	06:58	184			0.7			1		
Wednesday	19/08/2015	08:00							1		
Wednesday	19/08/2015	08:28	138	□	30	2.1			1		
Wednesday	19/08/2015	09:00							1		
Wednesday	19/08/2015	10:00							1		
Wednesday	19/08/2015	10:03	187						1		
Wednesday	19/08/2015	11:00							1		
Wednesday	19/08/2015	11:14	75						1		
Wednesday	19/08/2015	12:00							1		
Wednesday	19/08/2015	12:34	102						1		
Wednesday	19/08/2015	13:00							1		
Wednesday	19/08/2015	13:55	153	□	100	2.4	1.10 / 1.30 2:07 h		1		
Wednesday	19/08/2015	15:00							1		
Wednesday	19/08/2015	16:01	306						1		
Wednesday	19/08/2015	16:02				1.6				W8 ■	Stop<br>Bolus cancelled
Wednesday	19/08/2015	16:03				2.4			1	3.80 IU ▶	Start-up<br>Prime Insulin Pump
Wednesday	19/08/2015	18:24	175		20				1		

Legend/Caption

Hypoglycaemia	Hypoglycaemia symptoms	□ Before meal	■ After meal	✱ Asterisk
Before sport (23)	During sport (45)	After sport (24)	Stress (29)	Disease (31)

Day	Date	Time	Blood Glucose mg/dL	Blood Glucose symbols	Meal	Insulin 1	Insulin 2	Insulin 3	Basal	Insulin Pump	Comments
Wednesday	19/08/2015	18:25				0.6			1		
Wednesday	19/08/2015	18:51							1	W1	Cartridge low
Wednesday	19/08/2015	20:49	115	□	40	2.0			1		
Wednesday	19/08/2015	21:00							1		
Wednesday	19/08/2015	22:00							1		
Wednesday	19/08/2015	23:00							1		
Wednesday	19/08/2015	23:03								■	Stop
Wednesday	19/08/2015	23:04							1		Cartridge changed
Wednesday	19/08/2015	23:08							1	25.00 IU	Prime Insulin Pump
Wednesday	19/08/2015	23:11							1	25.00 IU	Prime Insulin Pump
Wednesday	19/08/2015	23:13							1	25.00 IU	Prime Insulin Pump
Wednesday	19/08/2015	23:15				1.0			1	▶ 18.30 IU	Start-up<br>Prime Insulin Pump
Thursday	20/08/2015	00:00				11.4			1		28.4
Thursday	20/08/2015	00:33	274			0.7			1		
Thursday	20/08/2015	00:34								■	Stop
Thursday	20/08/2015	00:35							1	▶ 11.50 IU	Start-up<br>Prime Insulin Pump
Thursday	20/08/2015	03:00							1		

Legend/Caption

Hypoglycaemia	Hypoglycaemia symptoms	Before meal	After meal	Asterisk
Before sport (23)	During sport (45)	After sport (24)	Stress (29)	Disease (31)

Day	Date	Time	Blood Glucose		Meal	Insulin			Basal	Insulin Pump	Comments
			mg/dL	Symbols		1	2	3			
Thursday	20/08/2015	04:00							1		
Thursday	20/08/2015	05:00							1		
Thursday	20/08/2015	06:00	178						1		
Thursday	20/08/2015	08:00							1		
Thursday	20/08/2015	08:11	82	[Before meal]	30				1		
Thursday	20/08/2015	08:12				1.8			1		
Thursday	20/08/2015	09:00							1		
Thursday	20/08/2015	09:53	122						1		
Thursday	20/08/2015	10:00							1		
Thursday	20/08/2015	10:33	91						1		
Thursday	20/08/2015	11:00							1		
Thursday	20/08/2015	11:13							1	[TBR decrease] 40%	dur 01:00 h
Thursday	20/08/2015	11:57	61	H					1		
Thursday	20/08/2015	12:00							1	[TBR decrease] 40%	
Thursday	20/08/2015	12:13							1		End of TBR
Thursday	20/08/2015	12:18	119						1		
Thursday	20/08/2015	13:00							1		

Legend/Caption

Hypoglycaemia	Hypoglycaemia symptoms	Before meal	After meal	Asterisk
Before sport (23)	During sport (45)	After sport (24)	Stress (29)	Disease (31)

Day	Date	Time	Blood Glucose mg/dL	Blood Glucose markers	Meal	Insulin 1	Insulin 2	Insulin 3	Basal	Insulin Pump	Comments
Thursday	20/08/2015	13:50	151	□	120	3.6	1.30 / 2.30 3:21 h		1		
Thursday	20/08/2015	15:00							1		
Thursday	20/08/2015	15:13	217						1		
Thursday	20/08/2015	17:10	296						1		
Thursday	20/08/2015	17:11				2.1				W8	Stop Bolus cancelled
Thursday	20/08/2015	17:12				1.0			1		Start-up
Thursday	20/08/2015	18:15	289						1		
Thursday	20/08/2015	18:16									Stop
Thursday	20/08/2015	18:17							1	10.00 IU	Start-up Prime Insulin Pump
Thursday	20/08/2015	18:29							1	250%	dur 01:00 h
Thursday	20/08/2015	19:29							1		End of TBR
Thursday	20/08/2015	20:25	61	H					1		
Thursday	20/08/2015	20:36	56	H					1		
Thursday	20/08/2015	20:53	106						1		
Thursday	20/08/2015	21:00							1		
Thursday	20/08/2015	21:58	174	□	30	2.2			1		
Thursday	20/08/2015	22:00							1		

Legend/Caption

Hypoglycaemia	Hypoglycaemia symptoms	Before meal	After meal	Asterisk
Before sport (23)	During sport (45)	After sport (24)	Stress (29)	Disease (31)

CASE 2 - CSII

2 weeks

Day	Date	Time	Blood Glucose mg/dL	Blood Glucose markers	Meal	Insulin 1	Insulin 2	Insulin 3	Basal	Insulin Pump	Comments
Thursday	20/08/2015	23:00							1		
Friday	21/08/2015	00:00				12.5			1		28.75
Friday	21/08/2015	01:25	271			1.3			1		
Friday	21/08/2015	03:00							1		
Friday	21/08/2015	04:00							1		
Friday	21/08/2015	05:00							1		
Friday	21/08/2015	05:51	124						1		
Friday	21/08/2015	07:07	132						1		
Friday	21/08/2015	07:45	111	□	30				1		
Friday	21/08/2015	07:46				2.1			1		
Friday	21/08/2015	08:00							1		
Friday	21/08/2015	09:00							1		
Friday	21/08/2015	10:00							1		
Friday	21/08/2015	10:07	68	H					1		
Friday	21/08/2015	10:28	79						1		
Friday	21/08/2015	11:00							1		
Friday	21/08/2015	11:01	128						1		

Legend/Caption

Hypoglycaemia	Hypoglycaemia symptoms	Before meal	After meal	Asterisk
Before sport (23)	During sport (45)	After sport (24)	Stress (29)	Disease (31)

Day	Date	Time	Blood Glucose mg/dL	Blood Glucose markers	Meal	Insulin 1	Insulin 2	Insulin 3	Basal	Insulin Pump	Comments
Friday	21/08/2015	11:59	185						1		
Friday	21/08/2015	12:00							1		
Friday	21/08/2015	13:00							1		
Friday	21/08/2015	13:56	136	□	120	3.9			1		
Friday	21/08/2015	15:00							1		
Friday	21/08/2015	16:15	76						1		
Friday	21/08/2015	17:42	130		30				1		
Friday	21/08/2015	17:43				1.1			1		
Friday	21/08/2015	19:25	138						1		
Friday	21/08/2015	20:38	165						1		
Friday	21/08/2015	21:00							1		
Friday	21/08/2015	21:32	173	□	70				1		
Friday	21/08/2015	21:33				4.1			1		
Friday	21/08/2015	22:00							1		
Friday	21/08/2015	23:00							1		
Friday	21/08/2015	23:52	103						1		
Saturday	22/08/2015	00:00				11.2			1		27.39

Legend/Caption

Hypoglycaemia	Hypoglycaemia symptoms	Before meal	After meal	Asterisk
Before sport (23)	During sport (45)	After sport (24)	Stress (29)	Disease (31)

Day	Date	Time	Blood Glucose mg/dL	Blood Glucose symbols	Meal	Insulin 1	Insulin 2	Insulin 3	Basal	Insulin Pump	Comments
Saturday	22/08/2015	03:00							1		
Saturday	22/08/2015	04:00							1		
Saturday	22/08/2015	04:44	283			1.7			1		
Saturday	22/08/2015	05:00							1		
Saturday	22/08/2015	08:00							1		
Saturday	22/08/2015	08:34	152	□	30	2.4			1		
Saturday	22/08/2015	09:00							1		
Saturday	22/08/2015	10:00							1		
Saturday	22/08/2015	10:20								■	Stop<br>Cartridge changed
Saturday	22/08/2015	10:26							1	25.00 IU	Prime Insulin Pump
Saturday	22/08/2015	10:28							1	25.00 IU	Prime Insulin Pump
Saturday	22/08/2015	10:29				1.0			1	▶<br>11.00 IU	Start-up<br>Prime Insulin Pump
Saturday	22/08/2015	10:54	129						1		
Saturday	22/08/2015	11:00							1		
Saturday	22/08/2015	12:00							1		
Saturday	22/08/2015	12:27	214			0.2			1		
Saturday	22/08/2015	13:00							1		

Legend/Caption

Hypoglycaemia	Hypoglycaemia symptoms	Before meal	After meal	Asterisk
Before sport (23)	During sport (45)	After sport (24)	Stress (29)	Disease (31)

Day	Date	Time	Blood Glucose mg/dL	Blood Glucose markers	Meal	Insulin 1	Insulin 2	Insulin 3	Basal	Insulin Pump	Comments
Saturday	22/08/2015	14:19	157	☐	60				1		
Saturday	22/08/2015	14:20				2.1			1		
Saturday	22/08/2015	15:00							1		
Saturday	22/08/2015	16:47	109						1		
Saturday	22/08/2015	17:37	122		30	0.9			1		
Saturday	22/08/2015	21:00							1		
Saturday	22/08/2015	21:28	124						1		
Saturday	22/08/2015	22:00							1		
Saturday	22/08/2015	22:30	191	☐	40	2.9			1		
Saturday	22/08/2015	23:00							1		
Sunday	23/08/2015	00:00				10.9			1		27.14
Sunday	23/08/2015	00:21	198						1		
Sunday	23/08/2015	03:00							1		
Sunday	23/08/2015	03:49	311						1		
Sunday	23/08/2015	03:50				2.0			1		
Sunday	23/08/2015	04:00							1		
Sunday	23/08/2015	05:00							1		

Legend/Caption

Hypoglycaemia	Hypoglycaemia symptoms	Before meal	After meal	Asterisk
Before sport (23)	During sport (45)	After sport (24)	Stress (29)	Disease (31)

Day	Date	Time	Blood Glucose mg/dL	Blood Glucose markers	Meal	Insulin 1	Insulin 2	Insulin 3	Basal	Insulin Pump	Comments
Sunday	23/08/2015	06:08	195						1		
Sunday	23/08/2015	08:00							1		
Sunday	23/08/2015	09:00							1		
Sunday	23/08/2015	09:13	76	□	30				1		
Sunday	23/08/2015	09:14				1.7			1		
Sunday	23/08/2015	10:00							1		
Sunday	23/08/2015	11:00							1		
Sunday	23/08/2015	12:00							1		
Sunday	23/08/2015	12:42	147						1		
Sunday	23/08/2015	13:00							1		
Sunday	23/08/2015	13:35	128	□	120				1		
Sunday	23/08/2015	13:36				3.8	1.20 / 2.60 4:00 h		1		
Sunday	23/08/2015	15:00							1		
Sunday	23/08/2015	16:06	230			1.0			1		
Sunday	23/08/2015	16:07	230						1		
Sunday	23/08/2015	17:46	217			0.5			1		
Sunday	23/08/2015	17:48								■ (stop)	Stop

Legend/Caption

Hypoglycaemia	Hypoglycaemia symptoms	□ Before meal	■ After meal	✱ Asterisk
Before sport (23)	During sport (45)	After sport (24)	Stress (29)	Disease (31)

Day	Date	Time	Blood Glucose mg/dL			Insulin 1	Insulin 2	Insulin 3		Insulin Pump	Comments
Sunday	23/08/2015	17:49							1	▶ 8.70 IU	Start-up Prime Insulin Pump
Sunday	23/08/2015	18:53	211			0.4			1		
Sunday	23/08/2015	21:00							1		
Sunday	23/08/2015	21:44	180	☐	30				1		
Sunday	23/08/2015	21:45	174	☐	30				1		
Sunday	23/08/2015	21:46				1.5			1		
Sunday	23/08/2015	22:00							1		
Sunday	23/08/2015	23:00							1		
Monday	24/08/2015	00:00				5.8			1		13.28
Monday	24/08/2015	00:14	259			1.1				■	Stop
Monday	24/08/2015	00:15							1		Cartridge changed
Monday	24/08/2015	00:26							1	▶ 22.20 IU	Start-up Prime Insulin Pump
Monday	24/08/2015	00:27				1.0			1		
Monday	24/08/2015	03:00							1		
Monday	24/08/2015	04:00							1		
Monday	24/08/2015	05:00							1		
Monday	24/08/2015	07:11	271			1.6				■	Stop

Legend/Caption

Hypoglycaemia	Hypoglycaemia symptoms	Before meal	After meal	Asterisk
Before sport (23)	During sport (45)	After sport (24)	Stress (29)	Disease (31)

Day	Date	Time	Blood Glucose mg/dL	Blood Glucose markers	Carbs	Insulin 1	Insulin 2	Insulin 3	Basal	Insulin Pump	Comments
Monday	24/08/2015	07:13							1	11.10 IU	Start-up<br>Prime Insulin Pump
Monday	24/08/2015	07:14							1	150%	dur 01:00 h
Monday	24/08/2015	08:00							1	150%	
Monday	24/08/2015	08:14							1		End of TBR
Monday	24/08/2015	08:15	234	□	30	2.1					Stop
Monday	24/08/2015	08:17							1	150%<br>15.00 IU	Start-up<br>dur 01:00 h<br>Prime Insulin Pump
Monday	24/08/2015	09:00							1	150%	
Monday	24/08/2015	09:17							1		End of TBR
Monday	24/08/2015	10:00							1		
Monday	24/08/2015	10:30									Stop

Legend/Caption

Hypoglycaemia	Hypoglycaemia symptoms	Before meal	After meal	Asterisk
Before sport (23)	During sport (45)	After sport (24)	Stress (29)	Disease (31)

Comb. Logbook

Day		00	01	02	03	04	05	06	07	08	09	10	11	12	13	14	15	16	17	18	19	20	21	22	23	
	BG	124								58 H 139				301		146 □		117		90		211	240 □		274	Med(10):170.0
Tuesday	CH															120				20			60			Tot.CH(3):200
11/08/2015	U													2.0		3.6						0.5	3.2		0.6	Tot.U(5):9.43
	Bas	0.45	0.45	0.45	1.0	0.9	0.8	0.8	0.8	0.6	0.5	0.4	0.5	0.8	0.7	0.7	0.75	0.75	0.75	0.75	0.75	5	0.6	0.7	0.6	= 17.08 =26.51
	Ev																			74 75						
	BG	306			174		141		138 □		310		228		103 □			75	143				175 □			Med(10):179.3
Wednesday	CH								30						140				20				34			Tot.CH(4):224
12/08/2015	U	0.6 1.0							2.4	1.0	0.4		0.6		4.1				0.9				2.4			Tot.U(9):13.87
	Bas	0.44	0.45	0.45	1.0	0.18	0.8	0.8	0.8	0.51	0.49	0.4	0.5	0.8	0.7	0.7	0.75	0.75	0.75	0.75	0.75	0.75	0.6	0.7	0.6	= 15.69 =29.56
	Ev																									
	BG					151				50 H 142			293		141 □			246	253			132	122 □	50 H	91 133	Med(12):150.3
Thursday	CH														80								60			Tot.CH(2):140
13/08/2015	U														2.8			1.2	0.7				3.0			Tot.U(4):7.7
	Bas	0.45	0.45	0.45	1.0	0.9	0.8	0.8	0.8	0.6	0.5	0.4	0.5	0.8	0.7	0.7	0.75	0.75	0.75	0.75	0.75	0.75	0.6	0.7	0.6	= 16.25 =23.95
	Ev																									
	BG	297			353	280	110			67 H 85			327		59 H 163		255	196	213			245		198 □		Med(14):203.4
Friday	CH																							40		Tot.CH(1):40
14/08/2015	U	1.6			1.7								2.3				1.9					1.5		2.0		Tot.U(6):11.0
	Bas	0.45	0.45	0.45	2.5	0.9	0.8	0.8	0.8	0.6	0.5	0.4	1.25	0.8	0.7	0.7	0.75	0.3	0.75	0.75	0.75	0.73	0.6	0.7	0.6	= 19.09 =30.09
	Ev																									
	BG			230					154 □			182	131		185 □			284		235	184	66 H	157	251	247	Med(12):192.2
Saturday	CH								30						140						20					Tot.CH(3):190
15/08/2015	U			1.2					2.5			0.3			2.4		1.0	0.4		1.1	1.0			1.4		Tot.U(9):11.3

Legend/Caption

H Hypoglycaemia	○ Hypoglycaemia symptoms	□ Before meal	■ After meal	* Asterisk

		00	01	02	03	04	05	06	07	08	09	10	11	12	13	14	15	16	17	18	19	20	21	22	23	
	Bas	0.45	0.45	0.44	1.0	0.9	0.8	0.8	0.8	0.6	0.5	0.4	0.5	0.8	0.7	0.65	0.75	0.75	0.75	0.73	0.75	0.75	0.6	1.75	0.6	= 16.64<br>+ =27.94
	Ev																									
	BG			158<br>165			101				103 □		139	131		120 □		236		262		181		151 □		Med(11):158.8
Sunday	CH										30					200				20				50		Tot.CH(4):300
16/08/2015	U										2.0					6.1		0.9		1.9				3.0		Tot.U(5):13.9
	Bas	0.45	0.45	0.45	1.0	0.9	0.8	0.8	0.8	0.6	0.5	0.4	0.5	0.8	0.7	0.7	0.75	0.75	0.75	0.53	0.53	0.75	0.6	0.7	0.6	= 15.86<br>+ =29.76
	Ev																									
	BG	267							315	267 □		192				95 □		75	152	228			254 □<br>246 □			Med(10):209.1
Monday	CH									30		20				100				20			30<br>30			Tot.CH(6):230
17/08/2015	U	0.7							2.0	2.1		1.1				2.8				2.2			1.9			Tot.U(7):12.8
	Bas	0.45	0.45	0.45	1.0	0.9	0.8	0.8	0.77	0.6	0.5	0.4	0.5	0.8	0.7	0.7	0.75	0.75	0.75	0.74	0.75	0.75	0.6	0.7	0.6	= 17.36<br>+ =30.16
	Ev																									
	BG			349						213 □		256	142		55 H<br>76 □			161	147		223		119 □			Med(10):174.1
Tuesday	CH									30			20		100								80			Tot.CH(4):230
18/08/2015	U			2.4<br>1.0						3.1		0.3	0.8		2.5				0.4		1.1		4.0			Tot.U(9):15.6
	Bas	0.45	0.45	0.44	1.0	0.9	0.8	0.8	0.8	0.6	0.5	0.6	0.5	0.8	0.7	0.7	0.75	0.75	0.75	0.75	0.73	0.75	0.6	0.7	0.6	= 16.56<br>+ =32.16
	Ev																									
	BG	95		266				184		138 □		187	75	102	153 □			306		175		115 □				Med(11):163.3
Wednesday	CH									30					100					20		40				Tot.CH(4):190
19/08/2015	U			1.5				0.7		2.1					2.4			2.4<br>1.6		0.6		2.0			1.0	Tot.U(9):14.3
	Bas	0.45	0.45	0.45	1.0	0.9	0.8	0.8	0.8	0.6	0.5	0.4	0.5	0.8	0.7	0.7	0.75	0.74	0.75	0.75	0.75	0.75	0.6	0.7	0.48	= 16.12<br>+ =30.42
	Ev																									
	BG	274						178		82 □	122	91	61 H	119	151 □		217		296	289		61 H	174 □			Med(15):151.8

Legend/Caption

H Hypoglycaemia	Hypoglycaemia symptoms	□ Before meal	■ After meal	* Asterisk

		00	01	02	03	04	05	06	07	08	09	10	11	12	13	14	15	16	17	18	19	20	21	22	23	
																						56<br>H<br>106				
Thursday 20/08/2015	CH									30					120								30			Tot.CH(3):180
	U	0.7								1.8					3.6				1.0<br>2.1				2.2			Tot.U(6):11.4
	Bas	0.44	0.45	0.45	1.0	0.9	0.8	0.8	0.8	0.6	0.5	0.4	0.2	0.8	0.7	0.7	0.75	0.75	0.74	0.73	0.75	0.75	0.6	0.7	0.6	= 17.0<br>=28.4
	Ev																									
	BG		271				124		132<br>111<br>□			68<br>H<br>79	128<br>185		136<br>□			76	130		138	165	173<br>□		103	Med(15):134.6
Friday 21/08/2015	CH								30						120				30				70			Tot.CH(4):250
	U		1.3						2.1						3.9				1.1				4.1			Tot.U(5):12.5
	Bas	0.45	0.45	0.45	1.0	0.9	0.8	0.8	0.8	0.6	0.5	0.4	0.5	0.8	0.7	0.7	0.75	0.75	0.75	0.75	0.75	0.75	0.6	0.7	0.6	= 16.25<br>=28.75
	Ev																									
	BG					283				152<br>□		129		214		157<br>□		109	122				124	191<br>□		Med(9):164.6
Saturday 22/08/2015	CH									30						60			30					40		Tot.CH(4):160
	U					1.7				2.4		1.0		0.2		2.1			0.9					2.9		Tot.U(7):11.2
	Bas	0.45	0.45	0.45	1.0	0.9	0.8	0.8	0.8	0.6	0.5	0.34	0.5	0.8	0.7	0.7	0.75	0.75	0.75	0.75	0.75	0.75	0.6	0.7	0.6	= 16.19<br>=27.39
	Ev																									
	BG	198			311			195			76<br>□			147	128<br>□			230<br>230	217	211			180<br>□<br>174<br>□			Med(12):191.4
Sunday 23/08/2015	CH										30				120								30<br>30			Tot.CH(4):210
	U				2.0						1.7				3.8			1.0	0.5	0.4			1.5			Tot.U(7):10.9
	Bas	0.45	0.45	0.45	1.0	0.9	0.8	0.8	0.8	0.6	0.5	0.4	0.5	0.8	0.7	0.7	0.75	0.75	0.74	0.75	0.75	0.75	0.6	0.7	0.6	= 16.24<br>=27.14
	Ev																									
	BG	259							271	234<br>□																Med(3):254.7
Monday 24/08/2015	CH									30																Tot.CH(1):30
	U	1.0<br>1.1							1.6	2.1																Tot.U(4):5.8

Legend/Caption

H Hypoglycaemia	Hypoglycaemia symptoms	□ Before meal	■ After meal	* Asterisk

	00	01	02	03	04	05	06	07	08	09	10	11	12	13	14	15	16	17	18	19	20	21	22	23	
Bas	0.36	0.45	0.45	1.0	0.9	0.8	0.8	0.77	0.58	0.5	0.2														= 7.48<br>+ =13.28
Ev																									

Legend/Caption

H Hypoglycaemia	O Hypoglycaemia symptoms	□ Before meal	■ After meal	* Asterisk

	Bedtime	Night	B. Breakfast	A. Breakfast	B. Meal	A. Meal	B. Dinner	A. Dinner	Statistics
n	12	16	23	23	20	27	27	6	154
MBG	215	217	151	172	136	188	165	155	173
(SD)	80	86	72	82	49	73	57	74	74
Hypos	0	0	3	2	2	0	3	1	11
Measurements *	0	0	0	0	0	0	0	0	0

	Nocturnal	Breakfast	Meal	Dinner	Bedtime	Statistics
n □		11	12	14		37
MBG □		152	133	179		156
(SD) □		62	30	45		50
n ■		0	0	0		0
MBG ■		0	0	0		0
(SD) ■		0	0	0		0
n(■ - □)		0	0	0		0
△BG (■ - □)						

		□	■
Frecuency of	11.0 (11.0)		
Number of tests	154	37	0
Mean bG	173 mg/dL	156 mg/dL	0 mg/dL
Standard Deviation	74 mg/dL	50 mg/dL	0 mg/dL
Highest Value	353 mg/dL	267 mg/dL	0 mg/dL
Lowest Value	50 mg/dL	76 mg/dL	0 mg/dL
Hypos	11		

Total number bolus
92

Mean total bolus
11.55 U

Code	Description	Code	Description	Code	Description
3	Snack	24	After exercise	72	Hyper warning
4	Sleep Time	29	Stress	73	Premenstrual
35	Oral medication	31	Disease	74	Other
36	User-defined	32	Menstruation	75	Exercise 1
20	Fast	45	During exercise	76	Exercise 2
23	Before exercise	71	Hypo warning	M	Manually entered value
